# High vaccination coverage, inadequate knowledge and high vector density: Findings from a community-based cross-sectional study on Japanese Encephalitis in Yangon, Myanmar

**DOI:** 10.12688/f1000research.21702.2

**Published:** 2020-06-30

**Authors:** Pyae Phyo Kyaw, Hemant Deepak Shewade, Nang Thu Thu Kyaw, Khaing Hnin Phyo, Htar Htar Lin, Aye Mon Mon Kyaw, Mg Mg Mya, Sein Thaung, Yan Naung Maung Maung

**Affiliations:** 1 Department of Medical Research, Ministry of Health and Sports, Yangon, 11191, Myanmar; 2 International Union against Tuberculosis and Lung Disease (The Union), Paris, 75006, France; 3 The Union South East Asia Office, New Delhi, 110016, India; 4 Karuna Trust, Bengaluru, 560041, India; 5 The Union Myanmar Country Office, Mandalay, 05021, Myanmar; 6 Expanded Programme on Immunization, Ministry of Health and Sports, Nay Pyi Taw, Myanmar; 7 Vector Borne Disease Control Program, Ministry of Health and Sports, Yangon, 11211, Myanmar

**Keywords:** JE vaccine, knowledge and perception, community-based survey, caregiver’s knowledge, SORT IT

## Abstract

**Background: **Japanese encephalitis (JE) is a mosquito-borne disease with high case fatality and no specific treatment. Little is known about the community’s (especially parents/guardians of children) awareness regarding JE and its vaccine in Yangon region, which bears the highest JE burden in Myanmar.

**Methods:** We conducted a community-based cross-sectional study in Yangon region (2019) to explore the knowledge and perception of parents/guardians of 1-15 year-old children about JE disease, its vaccination and to describe JE vaccine coverage among 1-15 year-old children. We followed multi-stage random sampling (three stages) to select the 600 households with 1-15 year-old children from 30 clusters in nine townships. Analyses were weighted (inverse probability sampling) for the multi-stage sampling design.

**Results:** Of 600 parents/guardians, 38% exhibited good knowledge of JE **, **55% perceived JE as serious in  children younger than 15 years and 59% perceived the vaccine to be effective **. **Among all the children in the 600 households, the vaccination coverage was 97% (831/855).

**Conclusion:** In order to reduce JE incidence in the community, focus on an intensified education program is necessary to sustain the high vaccine coverage in the community.

## Introduction

Japanese encephalitis (JE) is a zoonotic disease caused by Japanese encephalitis virus (JEV). JEV exists in a transmission cycle between mosquitoes, pigs and/or water birds and is transmitted to humans through bites from infected mosquitoes of the Culex species (mainly *Culex tritaeniorhynchus*) ^1^. JE usually presents as acute encephalitis syndrome (AES) and is confirmed by serology.

JE is a disease of public health importance as billions of people are at risk of getting infected by JEV and children below 15 years are more susceptible ^1^. A systematic review (2011) reported that 67,900 clinical cases of JE occur annually in 24 Asian and western Pacific countries despite the widespread availability of the vaccine, with approximately 13,600 to 20,400 deaths. While the overall incidence of JE is 1.8 per 100,000 per year in endemic countries, it is 5.4 among 1–15 year-old children ^2^. The infection can lead to severe complications with high case fatality. There is no specific treatment to date. JE is not easily prevented by protection from mosquito control and mosquito bites. Hence, vaccination is the most effective form of prevention ^1^. Globally, live attenuated SA 14-14-2 JE vaccine is the most commonly used JE vaccine. Vaccine efficacy is reported to be between 80% and 99% following single-dose vaccination and 98% or greater with two doses ^3^.

In Myanmar, *Culex tritaeniorhynchus* is the main JE vector ^4^. In 2012, there were only 14 confirmed JE cases, which increased to 151 in 2015 and more than 380 in 2016 and 2017, indicative of improved surveillance in the country ^5^.

In 2017, the Expanded Programme on Immunization (EPI) under the Department of Public Health carried out a nationwide JE vaccination catch-up campaign supported by the GAVI, WHO, the United Nations Children’s Fund and PATH. In November and December, nearly 14 million children (aged nine months to 15 years) were targeted and vaccinated with the WHO live attenuated (SA 14-14-2) JE vaccine. Alongside the campaign there were extensive advocacy and sensitization sessions provided to schools and communities ^6^. Since January 2018, immediately after the JE campaign, routine immunization in Myanmar has included JE live attenuated vaccine, given at the age of nine months together with the measles-rubella vaccine.

Adequate knowledge of JE and a positive perception of the JE vaccine are important for the adoption of preventive measures ^7^. In addition, high coverage of the JE vaccine in populations at risk of disease is required to reduce the JE cases because JE vaccination would not provide herd immunity ^8^. A study conducted in one township in northern Shan state in Myanmar (2018) showed the level of awareness of JE and its vaccine was low but the perception towards knowledge of JE was generally positive ^9^. The vaccination coverage was 93% among 391 study participants.

Little is known about the community’s (especially parents/guardians of children) awareness regarding JE and its vaccine in Yangon region, which bears the highest JE burden in the country ^6^. This data, alongside vaccination coverage data for children, may help the regional vector borne disease control (VBDC) programme and EPI to develop a new coordinated strategic plan to successfully reduce JE transmission in Yangon region.

Therefore, in Yangon region, we aimed to describe the i) knowledge and perception of the parents/guardians of children (1–15 years old) towards JE disease and vaccine, and ii) JE vaccine coverage among 1–15 year-old children.

## Methods

### Ethical statement

The Ethics Review Committee, Department of Medical Research, Myanmar (Ethics/DMR/2018/102EA/2019/028) and the Union Ethics Advisory Group of the International Union against Tuberculosis and Lung Disease (The Union), Paris, France (EAG 04/19) approved the study. Written informed consent for participation in the survey was taken from the parents/guardians of children aged 1–15 years old and the consent process was approved by the ethics committees.

### Study design

This was a community-based cross-sectional survey involving primary data collection.

### Study setting

Myanmar is located in the Southeast Asia region, neighboring Laos to the east, Bangladesh to the west, Thailand to the southeast, the Republic of China to the north and northeast and India to the northwest. Myanmar has a population of 51 million with an urban:rural population ratio of 30:70. The country has 14 states and regions including Nay Pyi Taw council territory. It consists of 74 districts, 330 townships, 398 towns, 3065 wards, 13,619 village tracts and 64,134 villages ^10^.

Yangon is the economic capital of Myanmar with four districts, 46 townships, 743 wards and 628 village tracts. The population of Yangon region is the highest in size when compared with other states and regions in the country. The urban:rural population ratio is 70:30 ^10^.

### Study population

The study population included parents/guardians of children (1–15 years old) living in Yangon region for the first objective and children (1–15 years old) for the second objective (February to June 2019).

### Sample size and sampling procedure

For the first two objectives, assuming that the prevalence of community awareness of JE and its vaccination among parents/guardians of children (1–15 years old) and vaccination coverage of children (1–15 years old) in Yangon being 50% with 5% precision at 95% CI, the calculated sample size was 384. Assuming a non-response rate of 5% and a design effect of 1.5, the final sample size was 600 households with children (1–15 years old) We used the conservative assumption of 50% prevalence as there is no previous data on community awareness and vaccination coverage in Yangon region and 5% non-response rate based on field experience.

**Figure 1.**
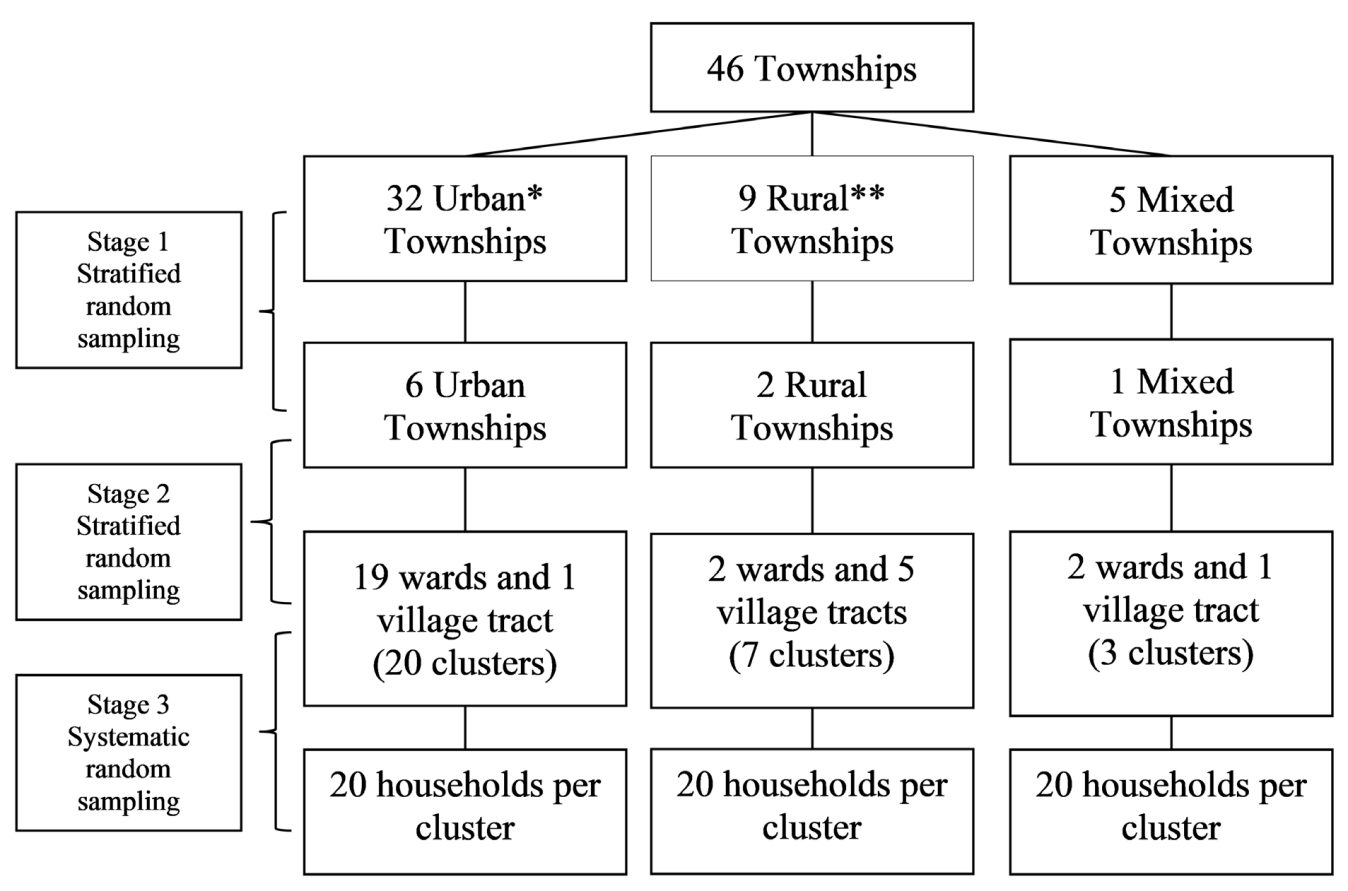
Multi-stage random sampling (three stage) to select households with 1–15 year-old children in the Japanese encephalitis survey, Yangon region, Myanmar (2019). *Predominantly urban (>70% urban population); **Predominantly rural (>70% rural population).

**Figure 2.**
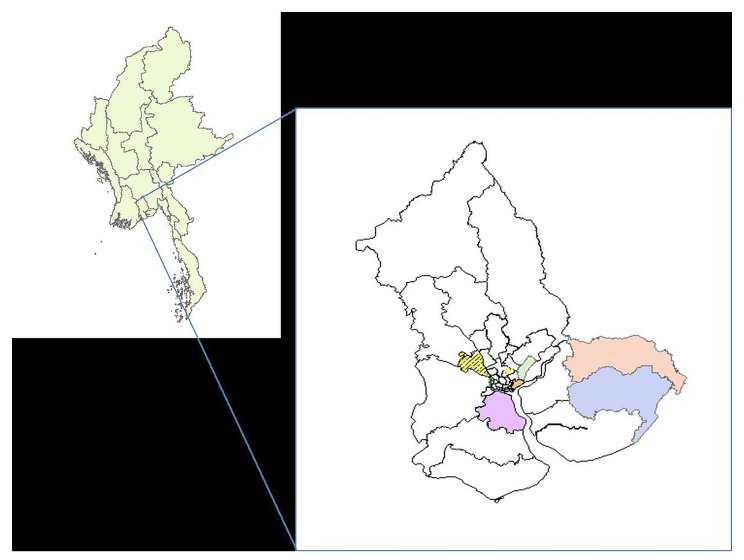
Randomly selected nine townships for the Japanese encephalitis survey, Yangon region, Myanmar (2019).

We used three stage random sampling to sample the 600 households with children (1–15 years) from 30 clusters (ward or village tract) in nine townships in Yangon region (see Figure 1 and Figure 2). In the first two stages, we used stratified random sampling and in the third stage, we used systematic random sampling. First, we randomly selected the nine townships from 46 townships maintaining a selection ratio of 1:5 in each strata (six predominantly urban, two predominantly rural and one mixed township) after stratifying them into urban (more than 70% urban population), rural (less than 30% urban population) and mixed (between 30% to 70% urban population) based on the classification used in population census in Myanmar ^10^. Then, in the second stage, thirty clusters were proportionately selected from six predominantly urban townships (20 clusters), two predominantly rural townships (7 clusters) and one mixed township (3 clusters) randomly after stratifying them into wards and village tracts in each selected township. Within each township, trained field assistants went directly to the general administration department for a list of the households and map of the selected wards/village tracts. The trained field assistants conducted systematic random sampling to select 20 households with children aged 1–15 years old within each cluster. The trained field assistants chose a random starting point using the map and then selected the first household. Then, they went to next household using the sampling interval until the required sample size was reached. Sampling interval was calculated by dividing the total number of household in the selected wards or village tracts by the required sample size for that selected wards or village tracts. If the selected household did not have any children aged 1–15 years old, field assistants went to the next adjacent household with a child in this age range. If the selected house was locked or there was no parent or guardian, field assistants followed the same procedure as above. In case the selected house was an apartment building, we selected one household randomly. We interviewed the parent or guardian of the child available at the time of survey, preferably the mother. There were no non-responses..

### Data collection and tools

During February-June 2019, at the selected households, field assistants conducted face to face interviews with parents/guardians using a pretested structured questionnaire (see Annex 1 and Annex 3, *Extended data*) ^11, 12^. The field assistant asked the questions verbally to the participants and completed the questionnaires. The structured questionnaires were tested during a pilot survey and were revised according to feedback received during the pilot survey.

The questionnaire consisted of four sections. The first section highlighted the socio-demographic information. The second section was comprised of 12 questions that assessed the knowledge of participants about JE disease and prevention (including vaccination). The third section assessed perception. The fourth section included information about vaccination status of the children. If the selected household had more than one child aged 1–15 years old, we asked the parent/guardian about the vaccination status of all the children in this age range. Vaccination status was based on parents’ or guardians’ recall. Field assistants also asked about the presence or absence of a vaccination card.

### Data management and analysis

Data from the survey forms were double-entered and validated using EpiData entry software (version 3.1, EpiData Association, Odense, Denmark). Data were analyzed using STATA (version 12.1, STATA Corp., College Station, TX, USA). There were no missing data in the study.

We provided weighted estimates as the analyses were weighted (inverse probability sampling) for the multi-stage sampling design. We used frequency and proportion to summarize the characteristics of the study participants. We assigned a knowledge score to each participant based on the number of correct or appropriate responses. Each appropriate answer was assigned one point and incorrect responses or “do not know” were assigned zero points. The scores were further dichotomized into poor or good (0–6 as poor and 7–12 as good). No overall score was calculated for perception. Vaccine coverage was calculated by the number of children that received JE vaccination (either during campaign or routine immunization) divided by the total number of children and presented as proportion and 95% confidence interval (CI).Odds ratios with 95% CI were estimated to determine the socio-demographic characteristics associated with good knowledge score and vaccination status using logistic regression. The characteristics with a p value of less than 0.2 in the unadjusted analysis were included in the multiple logistic regression.

## Results

The socio-demographic characteristics of 600 parents/guardians are presented in Table 1 ^13^. Among them, 1% were aged ≤ 20 years, 4% were aged >60 years and 74% were female. A total of 50% had a high school or graduate level education and 29% had a monthly family income of more than $285 USD.

**Table 1.** Socio-demographic characteristics of participants * in the Japanese encephalitis survey, Yangon region, Myanmar (2019) **.

Variable	N	(%) ***
**Total**	**600**	**(100.0)**
**Age in years**		
<=20	6	(1.0)
21–40	293	(48.8)
41–60	280	(46.6)
>60	21	(3.6)
**Sex**		
Male	155	(25.9)
Female	445	(74.1)
**Education level**		
Graduate	138	(22.9)
High School	165	(27.4)
Middle School	163	(27.2)
Primary School	105	(17.6)
Can read and write	24	(3.9)
Illiterate	5	(1.0)
**Occupation**		
Government staff	29	(4.9)
Private employee	186	(30.9)
Own business	150	(24.9)
Dependent	215	(35.9)
Others	20	(3.4)
**Type of family ^¥^**		
Nuclear	342	(56.2)
Extended	258	(43.8)
Family income per month (USD) *		
&95	8	(1.3)
95 – 189	101	(16.8)
190 – 285	316	(52.7)
>285	175	(29.2)

JE, Japanese encephalitis; USD, United States dollar; one USD = 1525 Myanmar Kyats. *Parent or guardian of the 1–15 year-old children available at time of survey, preferably mother. **Weighted estimates given taking into account the sampling design. *** Column percentage. ^**¥**^Nuclear: family which has father or mother with their children. Extended: family which has either grandfather, grandmother, uncle, or aunty in addition to the members in the nuclear family.

### Knowledge and perception

Overall, 37.6% exhibited good knowledge of JE. We have depicted the knowledge of respondents regarding cause, transmission, symptoms, prevention and treatment in Table 2. Among 600 parents/guardians, 49.3% had correct knowledge that JE is a fatal disease. Although 65.3% knew that the JE vaccine was available locally, only 26.8% correctly answered that vaccination is the most effective means to protect against JE.

It was found that 23% did not know the symptoms of JE, 16% responded incorrectly that JE has specific treatment and 58.8% responded that they did not know whether there is a treatment for JE or not. Participants responded that they used mosquito nets (31.3%), mosquito coils (14.1%) and spray or fumigation methods (10.7%) to avoid mosquito bites.

**Table 2.** Knowledge of participants * regarding cause, transmission, symptoms, prevention and treatment in the Japanese encephalitis survey, Yangon region, Myanmar (2019) **.

Question	Response	Total	
		n	(%) ***
**Total**		**600**	**(100.0)**
Have you ever heard about JE?	Yes	409	(68.2)
What is the cause of JE?	Virus	99	(16.5)
How does the disease get transmitted?	Mosquito	237	(39.5)
What are the common symptoms of JE?	High fever and convulsion	189	(31.5)
Is JE prevalent in children under 15 years?	Yes	264	(44.0)
Where does JE mostly prevail?	Rural areas and paddy fields	114	(19.0)
Are pigs the amplifier host?	Yes	129	(21.5)
Is JE a fatal disease?	Yes	296	(49.3)
Is there any specific treatment for JE?	No	151	(25.2)
Is JE a preventable disease?	Yes	331	(55.2)
Is the JE vaccine available locally?	Yes	392	(65.3)
How can one protect against JE?	JE vaccine	161	(26.8)

JE, Japanese encephalitis.

*Parent or guardian of the 1–15 year-old children available at time of survey, preferably mother.

**Weighted estimates given taking into account the sampling design.

*** Column percentage.

**Figure 3.**
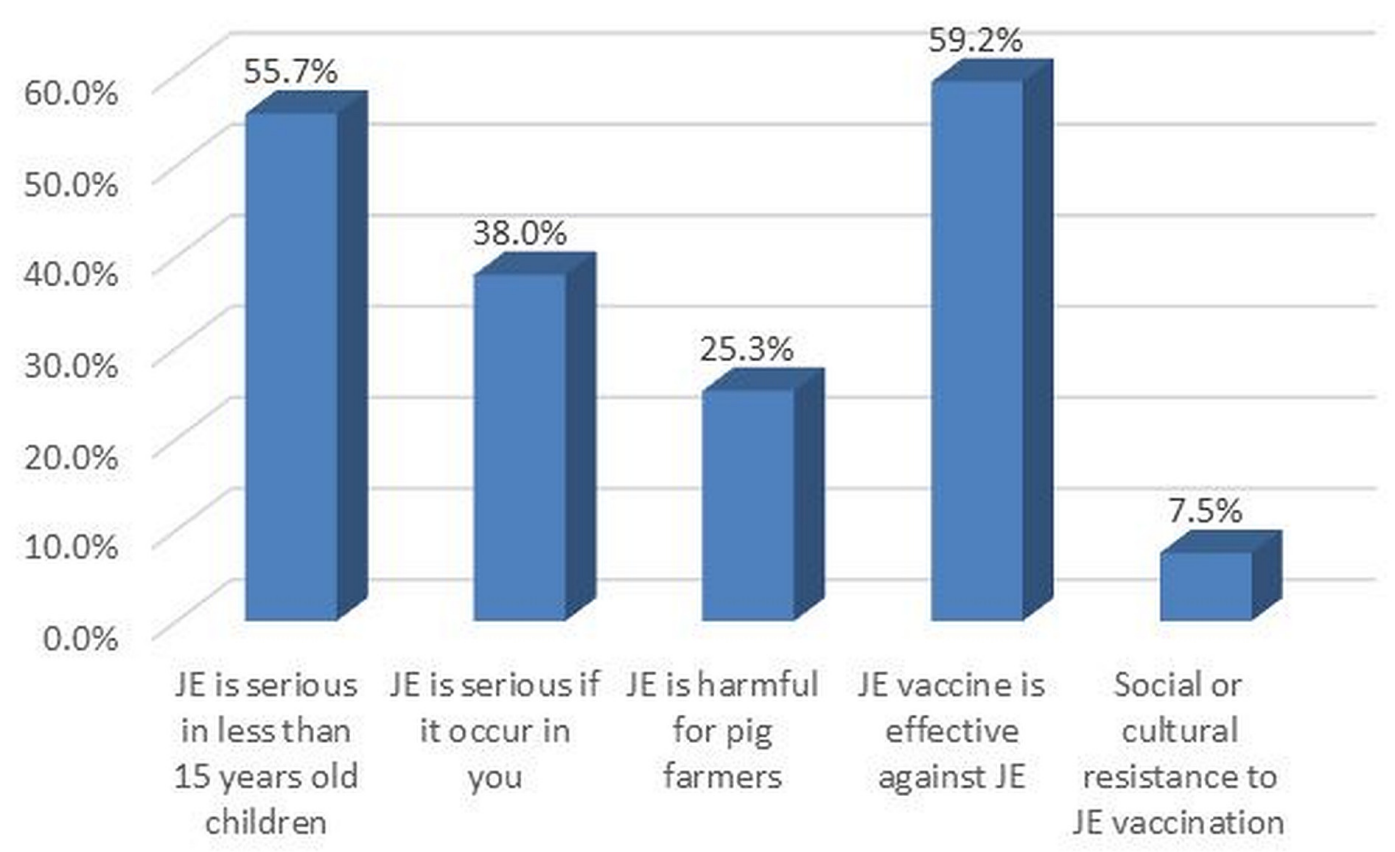
Perception of participants* in the Japanese encephalitis survey, Yangon region, Myanmar (2019) **. The variable “JE is serious if it occur in you” was included because sometimes adults think that some kinds of mosquito borne diseases such as Japanese encephalitis and Dengue are more serious in children and not serious when those diseases occur in them. Researchers in this study wanted to know whether participants had these perceptions. JE, Japanese encephalitis. Y axis as percentage (responded ‘yes’). *Parent or guardian of the 1–15 year-old children available at time of survey, preferably mother. **Weighted estimates given taking into account the sampling design.

**Figure 4.**
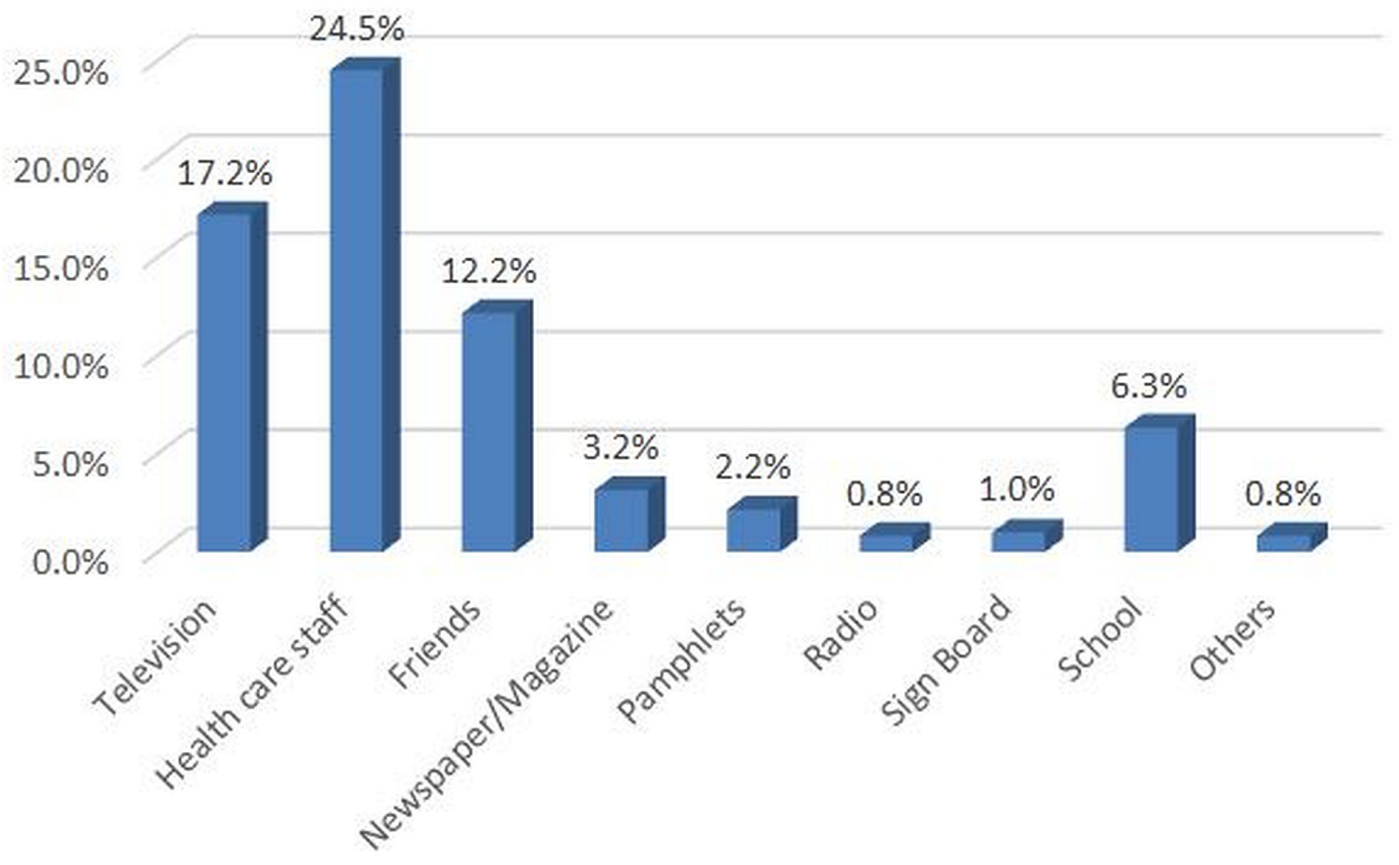
Source of information of participants* about Japanese encephalitis in the Japanese encephalitis survey, Yangon region, Myanmar (2019) **. Y axis as percentage (responded ‘yes’). *Parent or guardian of the 1–15 year-old children available at time of survey, preferably mother. **Weighted estimates given taking into account the sampling design.

Over half (55%) of participants perceived JE as serious in children younger than 15 years, 59% perceived the vaccine to be effective and 25% perceived JE to be harmful for pig farmers ( Figure 3). Health care staff (25%) and television (17%) were the main sources of information about JE disease ( Figure 4). The source of information on JE routine vaccination or campaigns was from school (35.1%), health worker visits (33.1%), announcements made using microphones in the neighborhood (13.9%) and volunteer visits (5.3%). The main vector of JE, *Culex tritaeniorhynchus*, had the highest proportion (41.2%) from entomological survey done in selected two townships out of nine townships in Yangon region.

### Vaccine coverage

Among all the children in the 600 households (n=855), 831 were vaccinated. The vaccination coverage was 97.2% (95% CI: 95.9-98.1). Of 831 vaccinated children, 423 (50.9%) were cross-checked through a vaccination card. Of 831 children, 516 (62.1%) received the vaccination during the JE campaign, 234 (28.2%) during routine immunization and 67 (7.8%) received the vaccine twice during both campaign and routine vaccination. Among the 24 children that did not receive vaccination, the main reasons were: parents or guardians did not realize the importance of JE vaccination (n=4), the child was sick at the time of immunization (n=3), the parents/guardians did not know about the JE vaccination (n=2) and travel (n=6).

### Factors associated with good knowledge score (≥7) and vaccination status

High level of education was the only variable significantly associated with good knowledge of JE **(≥7)**. ( Table 3). Good knowledge among respondents was significantly associated with the child being vaccinated (see Table 4). There was no association between socio-demographic characteristics of respondents and vaccination status.

**Table 3.** Factors associated with good knowledge (≥7 score) of JE among respondents in Yangon region, Myanmar (2019) **

Variables	Good knowledge of respondents				
	Crude OR	95% CI	Adjusted OR	95% CI	P value
**Sex** Male	1		1		
Female	1.371	0.859 – 2.189	1.288	0.765 – 2.170	0.340
**Education** Low level	1		1		
High level	1.980	1.333 – 2.942	2.332	1.532 – 3.550	<0.001 *
**Occupation** unemployed	1		1		
employed	0.759	0.509 – 1.131	0.623	0.394 – 0.985	0.043 *
**Family Type** extended	1		1		
nuclear	1.394	0.928 – 2.093	1.443	0.956 – 2.178	0.080
**Income** ≤189 USD	1		1		
>189 USD	1.011	0.622 – 1.644	0.932	0.563 – 1.543	0.786

JE, Japanese Encephalitis; *CI- Confidence Interval; **Weighted estimates given taking into account the sampling design

**Table 4.** Respondent level factors associated with vaccination against JE among children in Yangon region, Myanmar (2019) **.

Variables		Vaccination of children (yes)				
		Crude OR	95% CI	Adjusted OR	95% CI	P value
**Knowledge**	Poor	1		1		
	Good	3.500	0.943 – 12.981	3.978	1.220 – 12.964	0.022 *
**Age (year)**	≤20	1		1		
	21 to 40	6.310	0.578 – 68.791	5.744	0.669 – 49.264	0.111
	41 to 60	5.470	0.457 – 65.446	6.415	0.591 – 69.526	0.126
	>60	-	-	-	-	-
**Occupation**	unemployed	1		1		
	employed	2.195	0.663 – 7.266	2.583	0.678 – 9.832	0.164

JE, Japanese Encephalitis; CI- Confidence Interval * Statistically Significant **Weighted estimates given taking into account the sampling design

## Discussion

In this region-wide survey on JE and its vaccine coverage in Yangon, Myanmar, the majority of parents or guardians did not have good knowledge of JE. Perception of the seriousness of JE disease was poor in half of participants. Vaccination coverage was excellent.

### Strengths and limitations

Data were robust as double entry and validation minimized data entry errors. Only one attempt was made to visit each household, which may impact the generalizability of the results as the households with parents and guardians who were working during the survey visit might be missed. Half of parents/guardians did not produce a vaccination card (JE routine immunization or campaign) and recall bias cannot be ruled out. However, as JE vaccination is through an injection (subcutaneous) at a specific site (in the right upper arm) and the vaccination campaign was implemented only once in 2017, we do not think this is a serious limitation.

### Key findings and comparison with other studies

We found that parents/guardians had poor knowledge of JE. This finding is similar to the study conducted in a slum of Kolkata by Dasgupta *et al.* (2016), which showed that 56.7% of mothers had poor knowledge ^14^. Our study showed that 39.5% of participants knew that JE is caused by mosquito bites, which is higher than the 25.6% reported by Dasgupta *et al*. ^14^. In our study, the majority of participants showed a lack of knowledge about treatment of the disease, which is similar to a study from India (2015) by Ahmad *et al.* ^7^.

More than half of the participants perceived JE as serious in children younger than 15 years old, but they did not perceive pig farming as contributing to the threat of JE. This is similar to the study in India, where respondents did not perceive pig farming as contributing to the threat of JE ^15^. Possible reasons for poor knowledge and perception of participants was that communities did not think of JE as a possible threat like other vector-borne diseases such as dengue and malaria. In our study, high level of education had significant association with good knowledge and good knowledge of JE had significant association with vaccination of the child which is similar to a study by Dasgupta *et al* ^14^.

In our study, although only 55.2% of participants knew that JE is a preventable disease and 65.3% knew the most effective means to protect JE (vaccination), almost all parents/guardians vaccinated their children. Vaccination program against JE was started by EPI because of increased number of JE cases and death. To avoid death by JE, almost all parents/guardians got their children vaccinated even they did not know well about the nature of JE. It reflects their reliance on existing health care delivery systems and EPI. JE vaccine coverage was high in this study and an effective JE vaccination catch-up campaign by EPI might have contributed to the high vaccine coverage. That might be the reason for high coverage of vaccination in spite of low level of knowledge and perception about JE disease. Coverage is similar to the study from the district of Ambala, Haryana in north India, which demonstrated a high level (93.9%) of JE vaccine coverage in the study area ^16^. Similarly, JE vaccine coverage in Hsipaw Township, Northern Shan State, Myanmar was 93.0% in 2018 ^9^.

The most important vector is *Culex tritaeniorhynchus*, which feeds on vertebrate hosts, primarily pigs in preference to humans ^17^. Adult mosquito of *Culex tritaeniorhynchus* can spread JEV were collected from our entomological survey done in selected two townships out of nine townships in Yangon region which is similar with an entomological and vector bionomics study of JE transmission done in four villages of Letpadan Township, Bago region (Myanmar), showing a high proportion (69.8%) of *Culex tritaeniorhynchus* in areas with high pig farming ^18^.

### Implications

JE cases in Yangon region have declined in 2018 and 2019 compared to earlier years. In January – August 2019, there were only 244 AES cases and 12 confirmed JE cases ^19^. To sustain this decline, high vaccination coverage, health education on JE and effective vector control activity should be maintained. There is a possibility that the high coverage may not be maintained in the long term. This is because within 16–20 months of JE campaign, many were not aware of JE or its vaccine. Hence, steps should be taken in this direction.

Authorities should encourage retention of cards or records of vaccination so unvaccinated children can be identified and vaccinated in future.

## Conclusions

JE vaccination coverage was excellent in Yangon region, Myanmar, despite the majority of parents/guardians having poor knowledge and perception of JE disease, its prevention and vaccination. In order to reduce JE incidence in community, a focus on an intensified education program is necessary to sustain the high vaccine coverage in the community.

## Data availability

### Underlying data

Figshare: Annex 2. https://doi.org/10.6084/m9.figshare.10548623.v1 ^13^.

### Extended data

Figshare: Annex 1. https://doi.org/10.6084/m9.figshare.10552088.v1 ^11^.

Figshare: Annex 3. https://doi.org/10.6084/m9.figshare.11458437.v1 ^12^.

Data are available under the terms of the Creative Commons Zero “No rights reserved” data waiver (CC0 1.0 Public domain dedication).
